# Measuring the Evolution of Risk Communication Strategy for Health Authorities During the COVID-19 Pandemic: An Empirical Comparison Between China and the United States

**DOI:** 10.3389/ijph.2022.1604968

**Published:** 2022-11-28

**Authors:** Yue Yuan, Na Pang

**Affiliations:** Department of Information Management, Peking University, Beijing, China

**Keywords:** COVID-19, sentiment analysis, CDC, risk communication, quantitative analysis, news conference transcripts, NHC, topic analysis

## Abstract

**Objectives:** Investigate how the speech context of news conferences reveals the risk communication strategies for health authorities during COVID-19 and measure the evolution of those risk communication strategies.

**Methods:** We collected news conference transcripts concerning COVID-19 for the first quarter from the official websites of the Centers for Disease Control and Prevention (CDC) and the National Health Commission of the People’s Republic of China (NHC) in 2020. Quantitative analyses were conducted on the topics and emotions of transcripts to measure the evolution of risk communication strategy. A total of three types of analysis were carried out in our study: topic, sentiment, and risk communication evolution analyses.

**Results:** The trending topics and the number of these in the two institutions evolved with the infection status. The CDC and NHC maintained primarily neutral sentiment, while the non-neutral sentiment of the CDC swung more dramatically. Furthermore, the changing pattern of risk communication evolution for the CDC and NHC varied, where the latter had a more stable change routine.

**Conclusion:** Our study finds that the strategies could be measured by topic variation, emotional expressions, and confirmed cases. The CDC and NHC tend to adopt different risk communication strategies and have specific change routines facing the pandemic. In addition, our findings contribute to addressing the WHO research agenda for managing risk communication during the COVID-19 pandemic, which helps health authorities formulate and measure risk communication strategies.

## Introduction

The outbreak of COVID-19 constitutes an unprecedented public health crisis. As of 30 March 2022 [1], nearly 484 million confirmed cases, and more than six million deaths, have been reported. With the ever-increasing coronavirus infectors, the pandemic has not only had a devastating effect on public health services but also formed a so-called “infodemic” that generates a vast volume of accurate and inaccurate information, which increases the degree of public fear and uncertainty [2, 3]. Therefore, governments have raised significant concerns about risk communication to provide facts and practical guidelines for alleviating the public’s fears during the pandemic.

According to the World Health Organization (WHO) [4], the umbrella term “risk communication” is defined as “the real-time exchange of information between experts or officials and people who face the threat (from hazard) to their benefit.” The definition emphasizes the interaction among different roles [5] (e.g., governments, experts, journalists, and the public) where the discussion of topics (e.g., critical information about the emergency event) and interchange of emotions (e.g., fear or indifference) are often regarded as two critical components in the speech context of risk communication. A research question leading from this notion is measuring the speech context (i.e., topics and emotions) in risk communication. This question is essential in evaluating risk communication strategy but, to the best of our knowledge, has yet to be investigated. Current studies complement the field of risk communication research by focusing on the risk perceived and accepted by the public [6–13], while the analysis of content released by officials (e.g., governments/experts) lacks attention. Risk communication contains official release contents that express topics and emotions in different forms [14]. From this layer of concern, the risk communication strategy results from both topic selection and emotional expression [15–17]. This research thus investigates how the topics and emotions can be used to reveal the aforementioned strategy. Specifically, our research extracts topics and emotions from official release content and then reveals the relationship between them and risk communication strategy by answering the following questions:

(a) What topics and emotions are expressed in officially released content?
(b) What kind of risk communication strategy evolution could be revealed by analyzing topics and emotions from (a)?

To address the above questions, we analyzed the news conference transcripts from health authorities of China and the United States during the COVID-19 pandemic. Topics and emotions were acquired through quantitative methods to analyze the evolution of risk communication strategy. Furthermore, a comparison between China and the United States was conducted to summarize the similarities and differences when facing the virus.

## Methods

We proposed a quantitative analysis framework in which we acquired the news conference transcripts from the official websites of health authorities to measure the risk communication strategy using topic and sentiment analysis. The framework consisted of three steps: data collection, preprocessing, and analysis.

### Data Collection

We collected the news conference transcripts concerning the COVID-19 pandemic for the first quarter of 2020 from the official websites of the Centers for Disease Control and Prevention (“CDC”) of the United States and the National Health Commission of the People’s Republic of China (“NHC”) in 2020. We chose the above data for the following reasons:

(1) It was crucial for the government to pay attention to the risk communication in the early pandemic outbreak, which helped identify and address the uncertainty during the initial outbreak to perform the task of truth acknowledgment and emotional communication, as pointed out by Ernest et al. [18] together with Claire and Julie [19]. Therefore, we focused on the materials released from January to April 2020.
(2) China and the United States were dramatically affected in the early stage of the outbreak. Both CDC and NHC are crucial health authorities in two countries and held scheduled news conferences as an important way to conduct risk communication during the COVID-19 pandemic. Therefore, we chose the news conference transcripts they released on their websites to constitute our data source.

The details of the collected transcripts are listed in Table 1.

**TABLE 1:** Details of the news conference transcripts (the United States and China, 2020).

Institute name	CDC	NHC
Data source	https://www.cdc.gov/media/releases/2020/archives.html	http://www.nhc.gov.cn/xcs/fkdt/list_gzbd.shtml
No. of transcripts	17	63
No. of announcements	33	68
No. of questions	157	730
No. of answers	192	801
Time range	1st January 2020	1st April 2020
Language	English	Chinese
Country	The United States	The People’s Republic of China

The authoritative coronavirus case numbers should be acquired to conduct the analysis in the following step. We collected the confirmed case number from the WHO, where the exact confirmed case number and death number were recorded worldwide [1].

### Data Preprocessing

Since the language of NHC news transcripts was Chinese, this led to the conformity problem in the following data analysis step. Thus, we used the Baidu translation interface to translate Chinese NHC transcripts into English.

Since the news conferences are typically structured, the transcripts for the conference are usually divided into two parts: the announcement part and the question-answering (Q&A) part. The announcement part refers to the information briefing section on the news conference, while the Q&A part refers to the interaction section concerning the questions raised by journalists and other attendees. Here, the news conference transcripts for CDC and NHC have the announcement and Q&A parts. For analyzing the topics and sentiments in the next step, we focused on the Q&A sessions of the conferences. We focused on the Q&A part of the news conference because the Q&A part was more optimal than the announcement part in terms of information granularity and interactivity when revealing risk communication strategies. The announcement part delivered the main topic scope with coarse-grained information (e.g., overall pandemic situation) or specific events (e.g., certain infected cases) throughout the entire news conference, while the Q&A part usually included fine-grained information exchange and discussion under the topic scope of a news conference, which was more information-rich to be analyzed. Besides, the Q&A part of the news conference was more interactive than the announcement part, which accorded with the features (i.e., exchange of information) of risk communication.

Then, card sorting was carried out. Emerging from diverse fields, card sorting sheds light on how participants understand and organize concepts [20]. We extracted the questions in the transcripts and made small cards of these questions. We invited three experts to help us do the following card sorting to organize different questions into different topic categories. Experts organized cards into groups that they felt were appropriate to them. We summarized the groups classified by three experts into 20 topics to make each category as distinguishable as possible. The final topics are presented in Table 2, where the topic number and the description for each topic are listed.

**TABLE 2:** 20 topics of the news transcripts (the United States and China, 2020).

Topic number	Topic description	Topic definition
Topic #1	Testing	Information regarding different COVID-19 testing
Topic #2	Virus information	Names, origins and basic information about the novel coronavirus
Topic #3	Epidemic prevention	Different measures, guidance, and scenarios in preventing virus spread
Topic #4	Symptoms	Various symptoms for people who infected by the coronavirus
Topic #5	Diagnosis and treatment	Diagnosis and treatment methods for curation of SARS-COV2
Topic #6	Pandemic trends	Trends, development, and situation of the COVID-19 pandemic
Topic #7	Virus transmission	Different transmission media and infection modes for novel coronavirus
Topic #8	Travel restriction	Measures taken by different countries to restrict travel, border, and public transport
Topic #9	Curve control	Number of infections and effort for control the mortality rate
Topic #10	Drug development	Medicine specially developed for therapy towards SARS-CoV2 infectors
Topic #11	Vaccine development	The research and development process for vaccine
Topic #12	Health care workers	Conditions of health workers who fight the pandemic in the front
Topic #13	Supply support	Daily supplies and medical supplies
Topic #14	Vulnerable people	Conditions of older people and disabled people in the pandemic
Topic #15	Public information access	Transparency for public access to pandemic information
Topic #16	Information technology	5G, VR, artificial intelligence technologies in fighting against the pandemic
Topic #17	Financial support	Economic and financial support from the government
Topic #18	Mental health	Mental problems in the pandemic
Topic #19	Global pandemic	Pandemic happens in other places of the world and global cooperation
Topic #20	Others	Topic doesn’t belong to any of the above topics

### Data Analysis

We conducted topic, sentiment, and risk communication strategy analyses during the data analysis.

In topic analysis, we counted the number of topics for the CDC and NHC by week and used the topic heatmap to display the statistical results. Additionally, to acquire the ranking of these topics, the weekly statistics were aggregated and sorted by month. We then plotted the ranking results of different months for CDC and NHC to illustrate the monthly changes in topic rankings.

In sentiment analysis, we used Vader [21] to calculate the sentiment of answers for each question raised by the reporters at the news conferences of the CDC and NHC. The sentiments were measured by three types of emotional polarity: negative, positive, and neutral. The aforementioned sentiment was calculated proportionally, using the percentage to estimate the proportion of a certain emotional polarity among all emotional polarities. The proportion of polarity was depicted by week to show the sentiment change for CDC and NHC over time.

In the risk communication strategy analysis, the strategy was measured based on the results of topic and sentiment analyses. In detail, three indicators were used to depict the risk communication strategy: the number of confirmed cases, sentiment polarity, and topic number.

Peter Sandman believed risk could be measured by hazard and outrage and put forward the “risk = hazard + outrage” formulation [22]. According to different degrees of hazard and outrage, Peter Sandman divided risk communication strategy into four types: “health education, stakeholder relation,” “outrage management,” “crisis communication,” and “precautionary advocacy” outrage management. Unfortunately, he failed to give a quantitative evaluation method to measure the degree of hazard and outrage. Besides, he did not consider the dynamic changes in risk communication strategy. Inspired by the four kinds of risk communication strategies proposed by Peter Sandman, we employed a 2-dimensional coordinate system to depict the risk communication strategy and its change over time.

## Results

### Topic Analysis Results

**FIGURE 1:**
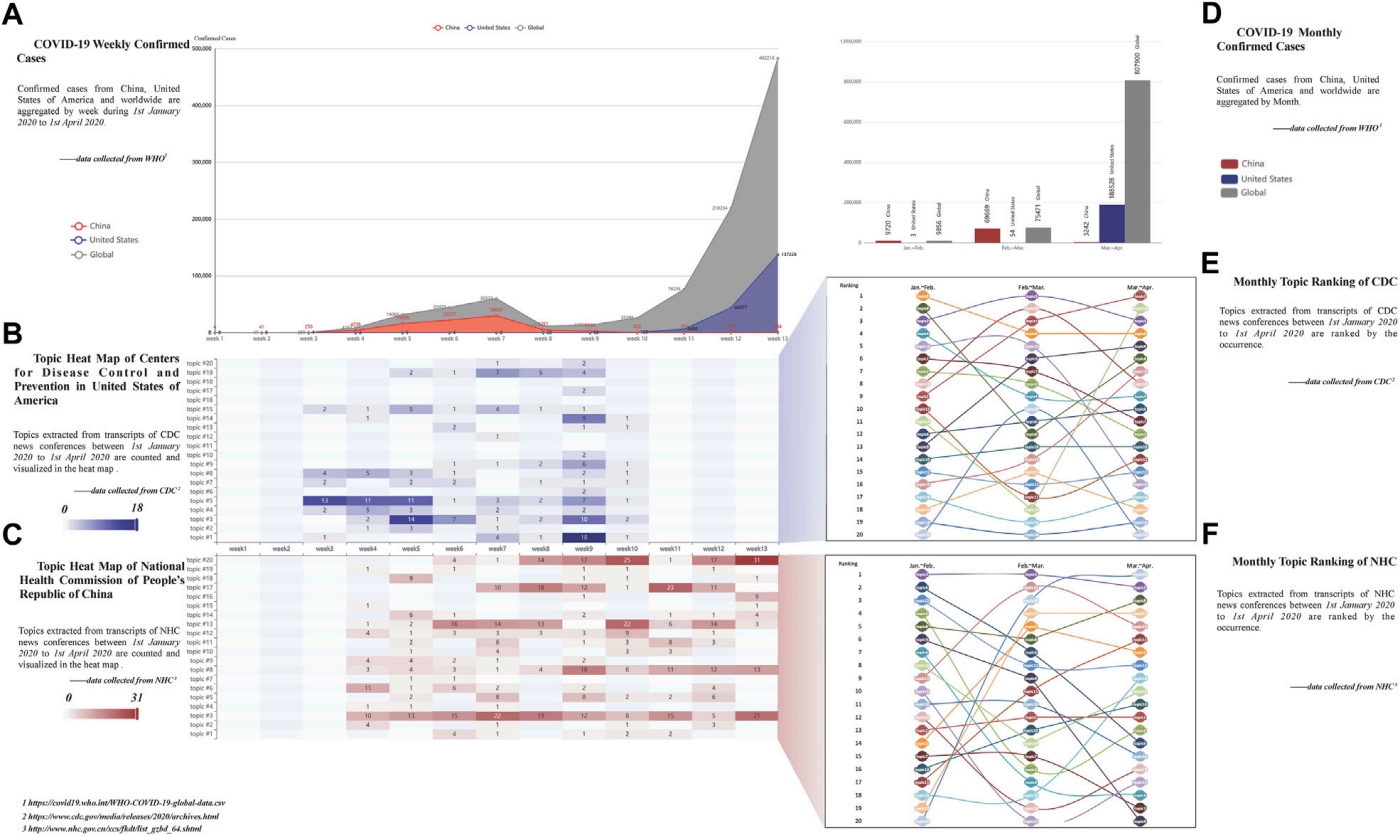
Results of topic analysis (the United States and China, 2020).

Figure 1 delineates the topic analysis results for news conference transcripts from the CDC and NHC, along with the statistics of confirmed COVID-19 cases in the United States and China during the initial pandemic period (from 1st January 2020 to 1st April 2020).

Figure 1 shows the temporal evolution of the topics and their relationships with the real-world infection status. The horizontal axes in Figures 1A–C represent time by weeks, while the vertical axes demonstrate the weekly confirmed cases for (a) and the number of different topics mentioned in news conferences during that week for (b) and (c). Topic heatmaps in Figures 1B,C can be reviewed jointly with Figure 1A in a vertical manner to observe the relationship between the change of topics and the change of confirmed cases over time. For instance, the CDC and NHC show quite different numbers of topic categories and occurrences in Figures 1B,C, with the worldwide and nationwide weekly confirmed cases changing simultaneously in Figure 1A.

In addition, based on Figures 1B,C, the number of topics changes through the different months. To exhibit the pattern of this variation, the ranking of topics and their monthly changes are illustrated in Figures 1E,F. The three columns in Figures 1E,F represent the occurrence ranking sequence of each topic during the first, second, and third months (i.e., January to February, February to March, and March to April). Meanwhile, the line connecting topics reveals the ranking changes. Figures 1E,F illustrate that the leading topics of the first, second, and third month keep changing for both CDC and NHC. This change could be detailed with the bar shown in Figure 1D, which displays the confirmed cases of the two countries by month. For example, the ranking of topic #1 (the topic about testing) for CDC in Figure 1E climbed from 9th to the top when the confirmed cases in the United States continued to rise from the first month to the third month.

In the result of the topic analysis, topics #3 (the topic about epidemic prevention), #5 (the topic about diagnosis and treatment), and #8 (the topic about travel restriction) are the most frequently mentioned both in CDC and NHC and exhibit good continuity during the initial pandemic period. Hence, the emotions of topics #3 (the topic about epidemic prevention), #5 (the topic about diagnosis and treatment), and #8 (the topic about travel restriction) are measured in further sentiment and risk communication strategy analyses.

### Sentiment Analysis

**FIGURE 2:**
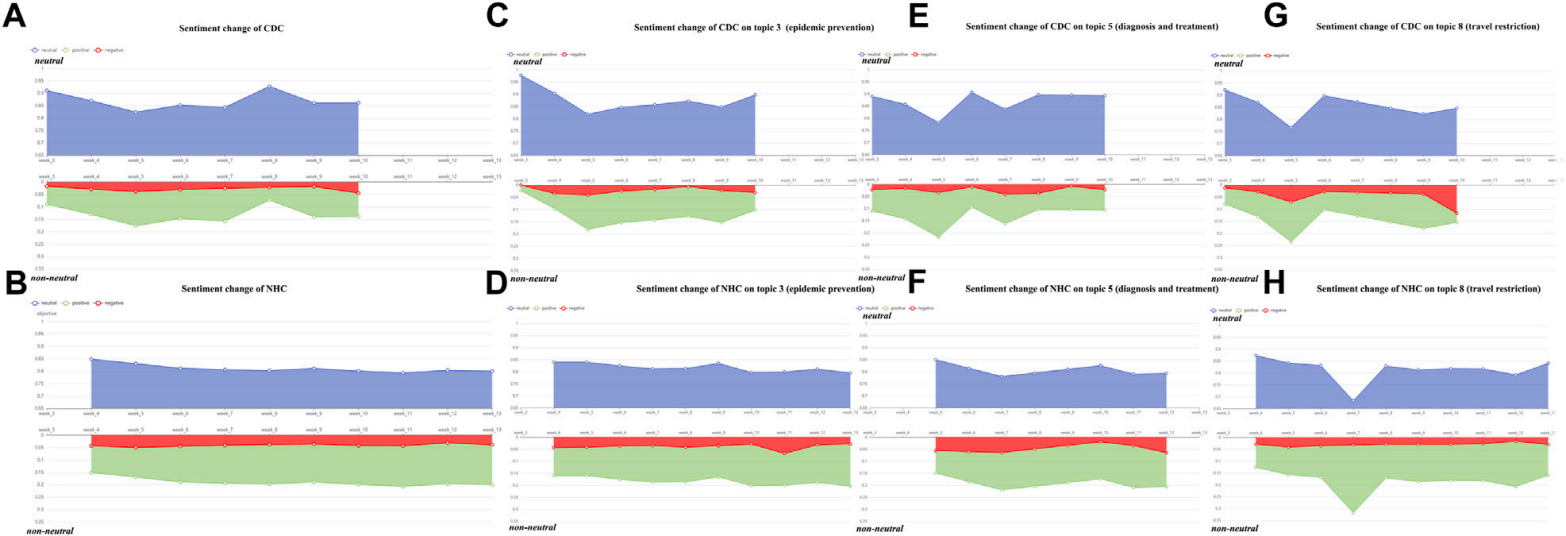
Results of sentiment analysis (the United States and China, 2020).

Sentiment analysis was conducted to quantify the emotional polarity of officials’ answers at the news conference. Figures 2A,B present the emotion polarity for the answers of the overall corpus for the CDC and NHC, while Figures 2C–H shows the polarity of topic #3 (the topic about epidemic prevention), #5 (the topic about diagnosis and treatment), and #8 (the topic about travel restriction) for the CDC and NHC, respectively.

The horizontal axes in the figures represent time by weeks, and the vertical axes are the percentage of two emotion polarities. The blue area in the figures depicts the intensity of neutral emotion, whereas the red and green areas describe emotions that are not neutral (i.e., positive and negative emotions). The size of the area reflects the percentage of different emotions. In Figure 2, the neutral part covers a larger area than the non-neutral part for both the CDC and NHC, whereas the proportion change of the non-neutral emotion of the CDC fluctuates more drastically than that of the NHC. In particular, week 5 is one of the most significant periods for the CDC, during which the proportion of non-neutral emotion peaks and the neutral emotion hits the lowest point. In contrast, the proportion of non-neutral emotion for NHC does not fluctuate at that time.

### Risk Communication Evolution Analysis

**FIGURE 3:**
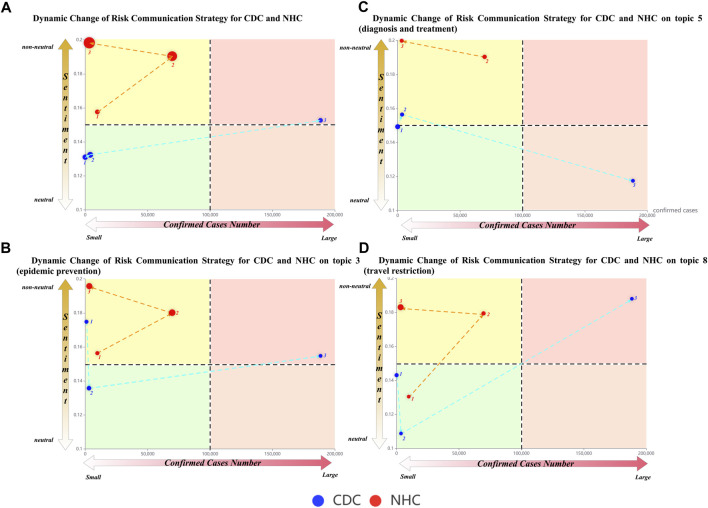
Results of risk communication strategy analysis (the United States and China, 2020).

During risk communication, CDC and NHC’s emotional tendencies and topic preferences evolve as the number of confirmed cases climbs. Such evolution can be depicted with the change route formed by dots and lines between them in a 2-dimensional manner. The change route for CDC and NHC can be regarded as the evolution of risk communication strategy, as illustrated in Figure 3.

In detail, Figure 3A illustrates general change routes for the CDC and NHC, while Figures 3B–D shows change routes for the CDC and NHC for topic #3 (the topic about epidemic prevention), #5 (the topic about diagnosis and treatment), and #8 (the topic about travel restriction), respectively. The confirmed case number is distributed on the horizontal axis, and the sentiment polarity is distributed on the vertical axis. The sentiment polarity is measured by the non-neutral level of the emotion, where the value on the vertical axis represents the sentimental level of the content answered by the officials. The dots depict risk communication strategy during a specific period. The occurrence number of the topic represents the size of the dots. The dot coordinate is determined by the confirmed case number and the sentiment polarity. The line among different dots represents the change direction of strategy for the CDC and NHC, which can be traced by the dotted arrow. Furthermore, each subfigure in Figure 3 is split into four colorful areas according to the intermediate value of both the confirmed case number (i.e., 100,000) and the proportion of non-neutral emotion (i.e., 0.15). Descriptions of the area are listed as follows:

(1) Green area: a small number of confirmed cases and relatively neutral emotion. The green area represents a “calm” strategy that officials communicate in an objective manner to tell the current risk of the pandemic is not high.
(2) Yellow area: a small number of confirmed cases and relatively non-neutral emotion. The yellow area represents a “precautionary” strategy that officials communicate in a non-neutral manner even if the current pandemic risk is not high.
(3) The orange area: a large number of confirmed cases and relatively neutral emotion. The orange area represents a “serious” strategy that officials communicate in a neutral manner, although the current pandemic risk is high.
(4) Red area: a large number of confirmed cases and relatively non-neutral emotions. The red area represents an “alert” strategy that officials communicate in a non-neutral manner since the current pandemic risk is high.

These areas can measure different change routes of risk communication strategy. In Figure 3, the proportion of non-neutral emotion of the NHC remains high from the first to the third month, while that of the CDC stays at a relatively low level in the first and second months and only rises slightly in the third month. However, the size of the dots representing the NHC has a tendency to get bigger, while that of the CDC stays the same. In addition, the dots in Figure 3 that represent the risk communication strategy of the NHC always remain in the yellow area, whereas those representing the strategy of the CDC in Figures 3B,C have crossed three different colored areas.

## Discussion

The key findings from the above analysis results are outlined below.

### Findings From Topic Analysis

The results of the topic analysis disclose the following findings. The trending topics for the CDC and NHC vary with their domestic and global pandemic situation. The topic variance for the two institutions is reflected by the difference that exists in the topic categories and occurrences that dynamically change over time (Figures 1B,C). We infer that such variance mainly results from the rapid development of the pandemic during the initial outbreak. Wicke et al. [23] and Chipidza et al. [24] proved that COVID-19 had a substantial effect on topics of public concern. Therefore, it is reasonable to speculate that the pandemic is driving the changes in the information released by CDC and NHC, which leads to the fact that topic trends for answers of CDC and NHC in Q&A are influenced accordingly. Through the results in Figure 1, the CDC demonstrates a clear preference for topics related to the prevention and control of the virus (topics #1, #3, and #5), while the NHC has a preference for pandemic control and supply support (topic #3, #8, and #13). The above findings can be regarded as important characteristics in risk communication for the CDC and NHC. Different topic preferences suggest that the two agencies have different focuses during the pandemic. Specifically, CDC experts answered many questions about the virus and pneumonia during Q&As, while NHC officials answered more about prevention and control. The functions of the two bureaucracies can help to explain the above differences: As one of the major operating components of the Department of Health and Human Services, CDC conducts critical science and provides health information that protects Americans from health threats. In comparison, the role of the NHC in responding to the pandemic covers a broad spectrum of public health (e.g., medicine supply, health, disease control, livelihood, etc.).

### Findings From Sentiment Analysis

Sentiment analysis reveals two interesting results regarding emotional expression in risk communication. First, the CDC and NHC responded to the questions in a relatively neutral tone. In general, we speculate this could be related to the identity of the people who responded to the reporter’s question. These people are usually experts and government officials who prefer to adopt a neutral or bureaucratic tone (Lavazza et al. [25]) when answering questions, which makes the result of sentiment analysis highly neutral. Second, although the neutral emotion occupies a high proportion for both the CDC and NHC, the proportion change of non-neutral emotions (i.e., negative or positive emotion) reveals the difference between the two institutions when facing the outbreak. The proportion change of non-neutral emotion for the CDC in risk communication is more dramatic than that of the NHC, indicating the different sentimental sensitivities of the two institutions when facing the same pandemic. Specifically, the negative or positive emotions of the NHC are expressed stably during the risk communication, while that of the CDC is more variable. For instance, in topic #3 (epidemic prevention), as the number of confirmed cases increased or decreased, the CDC and NHC showed different trends. The non-neutral sentiment proportion of NHC remains stable roughly between 0.15 and 0.2, while that of CDC changes more dramatically in the range of 0–0.2. The words used in the news conference to warn the public change with the increase of confirmed cases locally and globally. For example, CDC used the term “cautious” to warn the public in week 3. In contrast, such terms turned into “vigilant” in week 5, reflecting the rise of non-neutral sentiment proportion for arousing the awareness of precautions against the coronavirus. This means the CDC’s emotional changes in risk communication are more flexible than that of the NHC when they respond to changes in the pandemic.

### Findings From Evolution Analysis

The analysis of risk communication evolution reveals that the CDC and NHC have very different change routes of risk communication strategy. In particular, the pattern of the change route varies significantly. The NHC has a more stable change route than that of the CDC, with its dots mainly scattered in the yellow area, indicating that its risk communication strategy tends to be “precautionary,” where the non-neutral emotions (negative or positive) are likely to arouse the public attention to the potential risk. Therefore, we infer that the NHC has a high sensitivity in terms of the risk perception toward the (current and future) pandemic. In comparison, the change route for the CDC is much more volatile, which means the change degree of risk communication strategy is huge, and the change routes vary with the pandemic condition. For example, the attitude expressed by the CDC and NHC towards topic #8 (travel restriction) demonstrated different strategy changes: NHC showed an increase in precaution for travel and transportation (e.g., “aware of the virus spread by public transportation” in the first month, “take immediate action to cut the spread” in the second month, and “strictly prevent and control the personal travel” in the third month). In comparison, CDC was aware of the importance of travel restrictions only in the third month (e.g., alert to the public that 1/3 of the cases were infected by public transportation). The above findings indicate that the two institutions adopt different risk communication strategies when facing the pandemic. Their strategies are not always invariable. Instead, they have specific change routes which affected by the pandemic development. The change route could be measured by the variation of topics and emotions and the confirmed cases.

### Implications

Therefore, this study holds both theoretical and practical implications. For the theoretical implications, compared with previous research [26–31], this is the first that uses the official release news conference transcripts to quantitatively measure the evolution of risk communication strategy from topical and emotional perspectives. For the practical implications, the analysis results could be utilized to review the risk communication strategy for the CDC and NHC and provide a practical method for officials to evaluate their strategy.

### Limitations

However, the study still has several limitations. First, the number of the analyzed countries should be further expanded. More countries should be considered and analyzed to reveal the different genres of risk communication strategies. Second, the study’s data collection only covered the first quarter of 2020. An extended dataset incorporating longer time periods (e.g., 2020–2022) may lead to more intriguing findings. Third, we only conduct the quantitative correlation analysis from the topical and emotional perspectives to examine the risk communication strategy, while the causes and effects between the risk communication strategy and different variables (topics, sentiments, and the number of confirmed cases) remain unexplored. Therefore, the causation can be further explored by causal inference analysis. In addition, the “announcement” part of the news conferences, which contains the main topic scope of the new conference, is worth further coding by experts to supplement topic and sentiment analysis.

### Conclusion

In summary, this study presents a quantitative method to measure the evolution of risk communication strategy in the news conference transcripts of the CDC and NHC from topical and sentimental perspectives. Based on the results, we further explore the relationship between the pandemic situation and the change route of risk communication strategy. Our results suggest that the pandemic situation in the real world has influenced topic preference and emotional expression in risk communication. In addition, the risk communication strategy and its change route can be different according to the development of the pandemic.

The above findings contribute to addressing the WHO research agenda for managing risk communication in the COVID-19 pandemic, where the evaluation and analysis for risk communication and community engagement (RCCE) can benefit from our proposed method. For example, released information that includes producing and pre-test message templates to announce the infected case, preventive actions, and public health advice can be measured quantitatively to provide valuable recommendations and plans to the health authorities [32].
